# Gastrointestinal Tract Mycobacterial Infection in People Living With Human Immunodeficiency Virus (HIV)/Acquired Immunodeficiency Syndrome (AIDS) (PLWHA): Case Reports and Patterns of Endoscopic Involvement

**DOI:** 10.7759/cureus.104853

**Published:** 2026-03-08

**Authors:** Gabriella Cecília Vanin, José Vitor Santos-Oliveira, João Vitor Matachon Viana, Jose C Ardengh, Richard Calanca

**Affiliations:** 1 Infectious Diseases, Instituto de Infectologia Emílio Ribas, São Paulo, BRA; 2 Gastrointestinal Endoscopy, Hospital das Clínicas de Ribeirão Preto, Ribeirão Preto, BRA; 3 Diagnostic Imaging, Universidade Federal de Sao Paulo, São Paulo, BRA; 4 Digestive Endoscopy, Instituto de Infectologia Emílio Ribas, São Paulo, BRA

**Keywords:** diagnostic imaging, gastro, hiv, mycobacterium infection, upper endoscopy

## Abstract

Opportunistic infections result from infections caused by bacteria, mycobacteria, viruses, fungi, protozoa, or helminths, potentially leading to severe disease and death. These infections are a significant cause of morbidity and mortality among individuals with profound immunosuppression, such as people living with human immunodeficiency virus (HIV)/acquired immunodeficiency syndrome (AIDS) (PLWHA) and solid organ transplant recipients. Mycobacterial infections, including those caused by *Mycobacterium tuberculosis* and nontuberculous mycobacteria (NTM), particularly the *Mycobacterium avium* complex (MAC), are responsible for potentially severe and disseminated disease. In this article, we report three cases from a public teaching hospital in the state of São Paulo, Brazil, involving the evaluation of PLWHA presenting with nonspecific gastrointestinal symptoms and the diagnostic workup for gastrointestinal tract (GIT) mycobacterial infection, with a focus on endoscopic imaging findings and their different patterns of involvement.

## Introduction

Opportunistic infections are an important cause of morbidity and mortality among populations living with some degree of immunocompromise, such as people living with human immunodeficiency virus (HIV)/acquired immunodeficiency syndrome (AIDS) (PLWHA), solid organ transplant recipients, and individuals receiving immunosuppressive therapy [1,2]. This susceptibility is attributed to the interaction between impaired immunity and alterations in the individual's commensal flora [1]. In PLWHA, opportunistic infections are directly correlated with CD4 T-lymphocyte counts, and the risk increases as CD4 levels decline, with particularly high susceptibility among those with CD4 counts below 50 cells/mm³ [1-3]. In this CD4 range, the risk of severe infections such as disseminated *Mycobacterium avium *complex (MAC) disease, cytomegalovirus (CMV) retinitis, primary central nervous system lymphoma, and disseminated histoplasmosis is markedly increased [2].

One of the sites that can be affected by opportunistic infections is the gastrointestinal tract (GIT) [4]. Diagnosis is challenging and requires a high index of clinical suspicion and the combination of multiple diagnostic methods [5]. Many pathogens can cause opportunistic GIT infections, including bacteria, mycobacteria, viruses, fungi, protozoa, and helminths [2,6-8]. Among bacteria, non-typhoidal *Salmonella*, *Shigella*, *Campylobacter*, and diarrheagenic *Escherichia coli *can be highlighted [2]. Among viruses, CMV, herpes simplex virus (HSV), and Epstein-Barr virus (EBV) are noteworthy [4,6]. Among fungi, *Candida albicans *and *Cryptococcus *spp. are prominent [4,6,9]. Among protozoa, *Cryptosporidium *spp., *Giardia intestinalis*, *Blastocystis *spp., *Cystoisospora belli*, *Cyclospora cayetanensis*, and microsporidia should be mentioned [2,7]. Among helminths, *Strongyloides stercoralis *deserves particular attention [8]. Finally, among mycobacteria, infections caused by tuberculous mycobacteria and nontuberculous mycobacteria (NTM) are included [2,6].

Mycobacteria, grouped within the genus *Mycobacterium*, are Gram-positive, acid-fast bacilli (AFB) and comprise approximately 188 species [10]. There are several ways to classify mycobacteria, whether by clinical groups or phylogenetically, although no single classification has been universally accepted in the literature [10-13]. Considering the clinical classification, they can be divided into four major clinical groups: the *Mycobacterium tuberculosis *complex (MTBC), the *Mycobacterium leprae *complex, *Mycobacterium ulcerans*, and NTM, within which the MAC stands out [11-16].

The MTBC includes a group of closely related species, such as *Mycobacterium tuberculosis *sensu stricto, *Mycobacterium africanum*, *Mycobacterium bovis*, *Mycobacterium orygis*, *Mycobacterium microti*, and *Mycobacterium caprae*, among others [5,13]. The *Mycobacterium leprae *complex is composed of *Mycobacterium leprae *and *Mycobacterium lepromatosis *[15]. NTM consist of species not belonging to the two aforementioned complexes and can be divided into rapidly growing mycobacteria, such as the *Mycobacterium abscessus *complex (MABC), *Mycobacterium fortuitum *complex, and *Mycobacterium chelonae*, and slowly growing mycobacteria, such as *Mycobacterium kansasii*, *Mycobacterium marinum*, *Mycobacterium gordonae*, *Mycobacterium ulcerans*, and *Mycobacterium xenopi*; among these, a clinically important subgroup is the MAC, which includes *Mycobacterium avium*, *Mycobacterium intracellulare*, and *Mycobacterium intracellulare *subsp. *chimaera *[12,16].

A literature review was conducted in the main scientific databases, namely, MEDLINE, LILACS, and SciELO, using the descriptors "mycobacteriosis" or "mycobacterium" and "endoscopy" and "HIV", including articles published between 2019 and 2023 in Portuguese and English and available in full text. After this review, it was found that there is no comprehensive approach in the scientific literature addressing GIT mycobacteriosis, with most publications consisting of case reports and focusing on isolated mycobacterial species.

Therefore, the aim of this case series is to describe three cases of GIT mycobacteriosis in PLWHA, with an emphasis on endoscopic patterns of involvement and diagnostic challenges.

## Case presentation

Case 1

**Figure 1:**
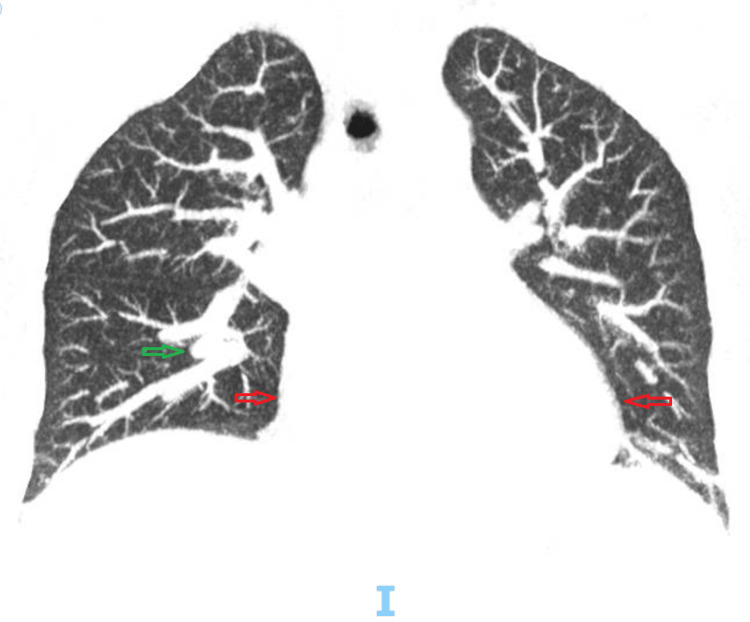
Coronal chest CT scan demonstrating diffuse thickening of the bronchial walls, suggestive of inflammatory bronchopathy, predominantly affecting the lower lobes (green arrows). Associated findings include mucoid impaction and cardiomegaly (red arrows) CT: computed tomography

**Figure 2:**
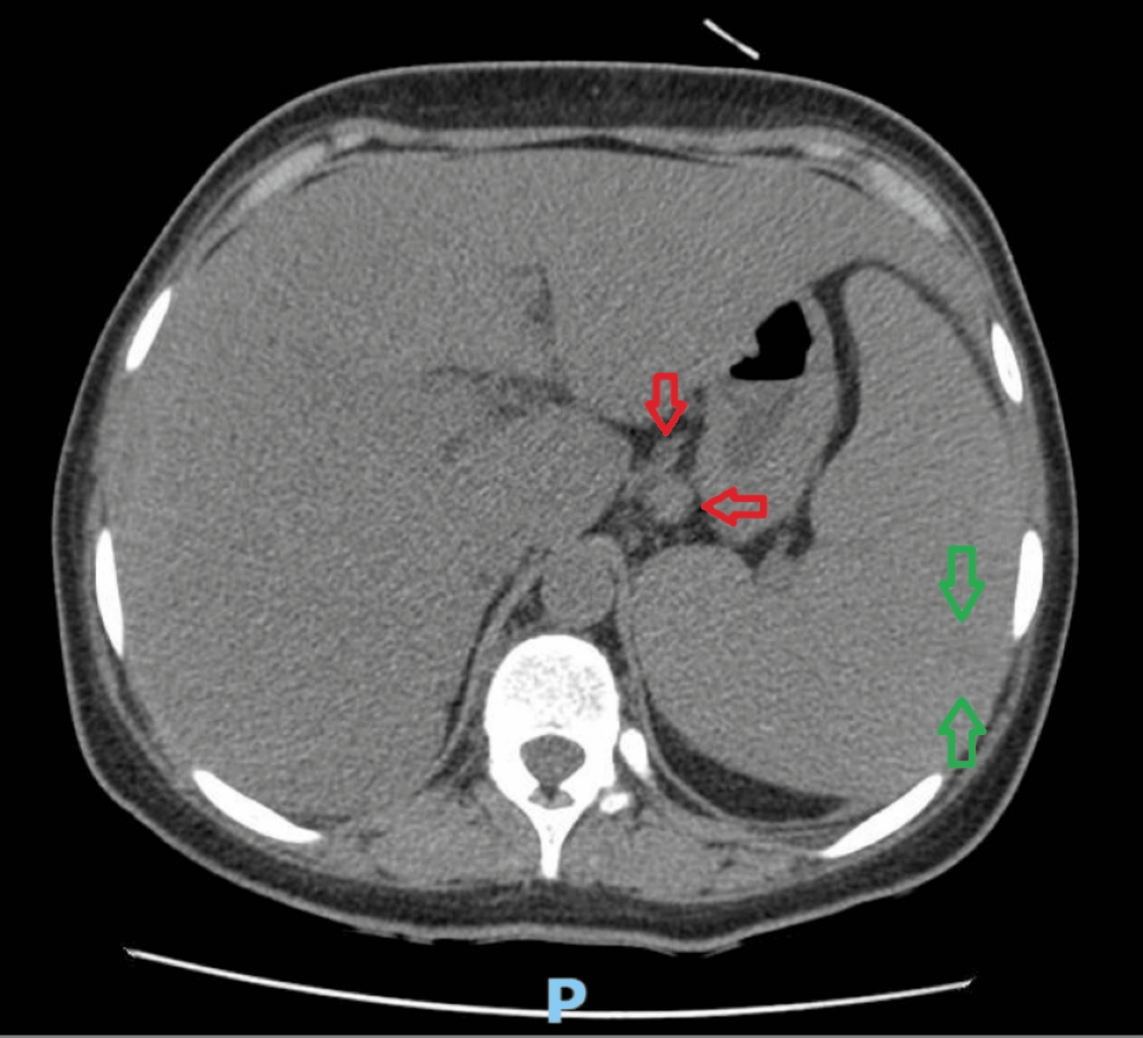
Axial contrast-enhanced CT scan of the upper abdomen (arterial phase) revealing multiple enlarged retroperitoneal, mesenteric, and iliac lymph nodes (red arrows), in addition to splenomegaly with probable infarcted areas (green arrows) characterized by cuneiform hypervascular patterns CT: computed tomography

A 27-year-old patient, born and residing in São Paulo, with a diagnosis of vertically transmitted HIV infection since the age of two, was lost to follow-up at the time of presentation. She had an HIV viral load (HIV-VL) of 279,831 copies (5.45 log) and a CD4 T-cell count of 16 cells/mm³, as well as severe bulimia. She presented to the emergency department (ED) with complaints of dry cough, dyspnea, daily fever, and weight loss for approximately two months. Concomitantly, she reported a progressive gastrointestinal condition over the previous seven months, including watery diarrhea without pathological contents, solid food dysphagia, poor appetite, and postprandial fullness. Laboratory tests revealed pancytopenia (hemoglobin 6 g/dL, leukocytes 2,100/mm³, and platelets 142,000/mm³). Imaging studies (chest and total abdominal computed tomography (CT)) showed inflammatory bronchopathy, particularly in the lower lobes, cardiomegaly, retroperitoneal, mesenteric, and iliac lymphadenopathy, as well as splenomegaly with areas suggestive of splenic infarctions and a small amount of free fluid in the peritoneal cavity (Figures 1-2). The patient was admitted for further investigation, and differential diagnoses included mycobacterial infection (MAC and MTBC), histoplasmosis, CMV infection, and diarrhea caused by opportunistic pathogens in PLWHA or parasitic infections.

During the diagnostic workup, all blood cultures (six serum cultures for mycobacteria and 15 aerobic serum cultures) were negative. Sputum samples were tested for AFB smear, mycobacterial culture for tuberculosis, and the Rapid Molecular Test for Tuberculosis (Xpert MTB/RIF), all of which were negative or not detected. Bronchoalveolar lavage was negative for AFB smear, Xpert MTB/RIF, fungal investigation (with culture positive for *Candida albicans*), and Gram stain. Gastric lavage showed a negative AFB smear and a negative culture for tuberculosis. A bone marrow aspirate was performed, and cultures were negative for fungi and mycobacteria. In addition, echocardiography was within normal limits with no vegetations; serum cryptococcal antigen was nonreactive; and histoplasmosis investigation with serum immunodiffusion and polymerase chain reaction (PCR) showed inconclusive and negative results, respectively.

All stool tests were negative: four samples for fresh smear larval examination; three samples for aerobic culture (no growth of enteropathogenic flora), direct microscopy for amoebae, leukocyte testing (with numerous red blood cells observed), and opportunistic pathogen screening (*Cryptosporidium *sp. and *Isospora belli*); parasitological examinations (Ritchie technique for helminths and protozoa; Lutz/Hoffman, Pons, and Janer techniques for helminths and protozoa; and modified Rugai method); two samples for rapid testing for rotavirus, enzyme-linked immunosorbent assay (ELISA) for *Cryptosporidium *sp., *Blastocystis hominis *detection, and fresh smear and macroscopic examination for helminths; and one sample for rapid adenovirus testing, *Schistosoma mansoni *egg count, direct examination for helminths and protozoa, and eosinophil detection. Glutamate dehydrogenase (GDH) tests were positive; however, *Clostridioides difficile *toxins A and B were negative. Fecal occult blood testing was positive in three samples.

An upper gastrointestinal endoscopy (UGE) was performed in August 2021 (images unavailable), revealing esophageal candidiasis and distal ulcerative esophagitis. Histopathological examination showed the esophagus with a fibrinoleukocytic plug and granulation tissue, consistent with the base of an ulcerated lesion, with rare cytopathic changes suggestive of viral activity. AFB staining using Ziehl-Neelsen and fungal stains using periodic acid-Schiff (PAS) and Grocott were negative. Empirical treatment for CMV with ganciclovir was initiated. Subsequently, immunohistochemistry (IHC) results were negative for CMV and human herpesvirus type 8 (HHV-8) antigens.

A follow-up UGE performed in September 2021 (images unavailable) showed a shallow ulcer at the esophagogastric junction, covered by a thin fibrin layer and surrounded by hyperemia. Histopathology at this time demonstrated an ulcerated lesion in the squamocolumnar junction mucosa of the foveolar type, associated with an early granulomatous pattern. AFB and fungal studies remained negative, with no evidence of viral infection or cellular atypia. Esophageal immunohistochemistry was positive for HSV and negative for CMV and mycobacteria. Treatment with acyclovir was initiated for the herpetic lesion. In addition, due to persistent febrile episodes, empirical therapy with the RHZE regimen (rifampin, isoniazid, pyrazinamide, and ethambutol) plus clarithromycin was started, targeting MAC and MTBC.

**Figure 3:**
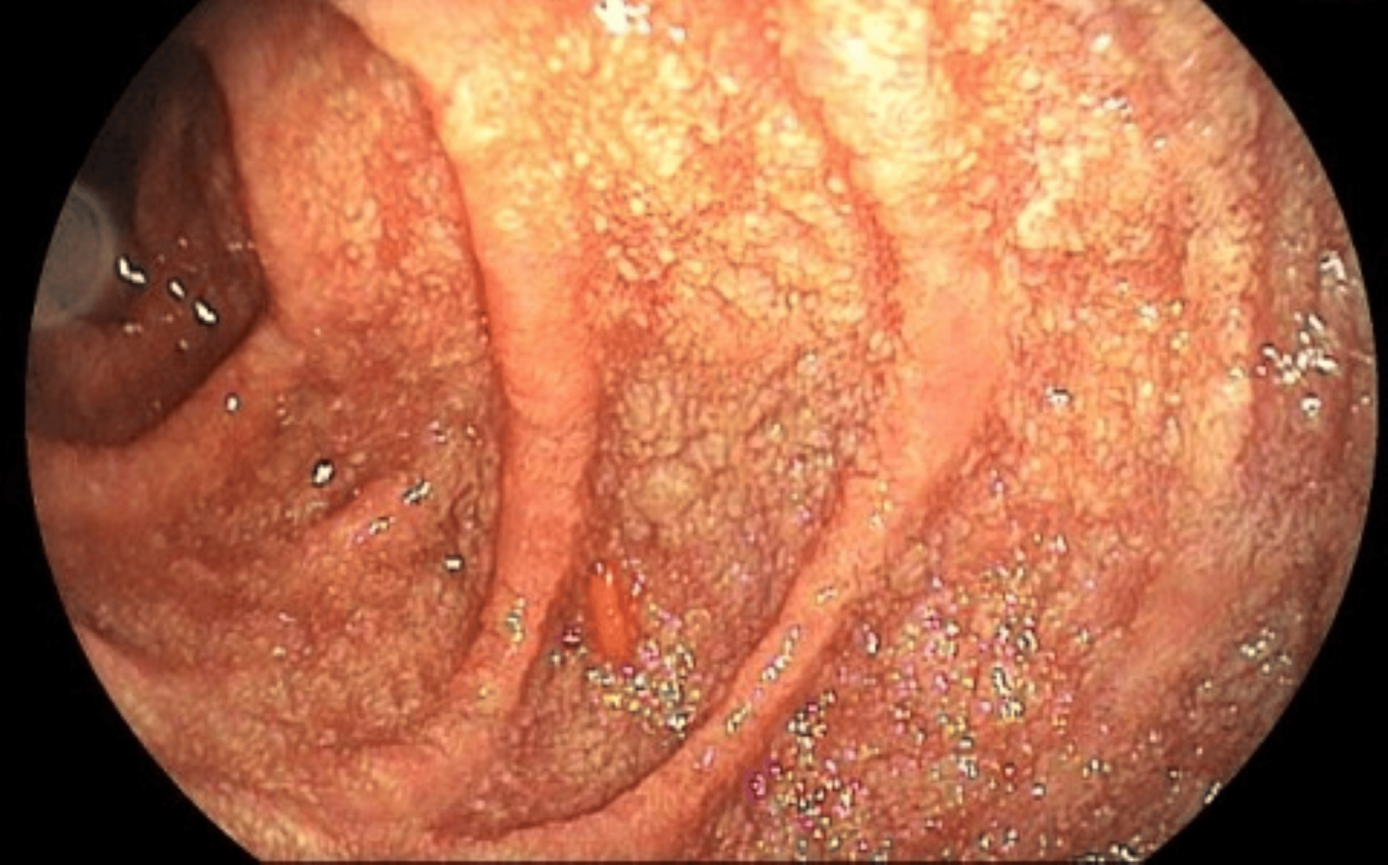
Duodenal mucosa with irregular, granular, and whitish appearance

**Figure 4:**
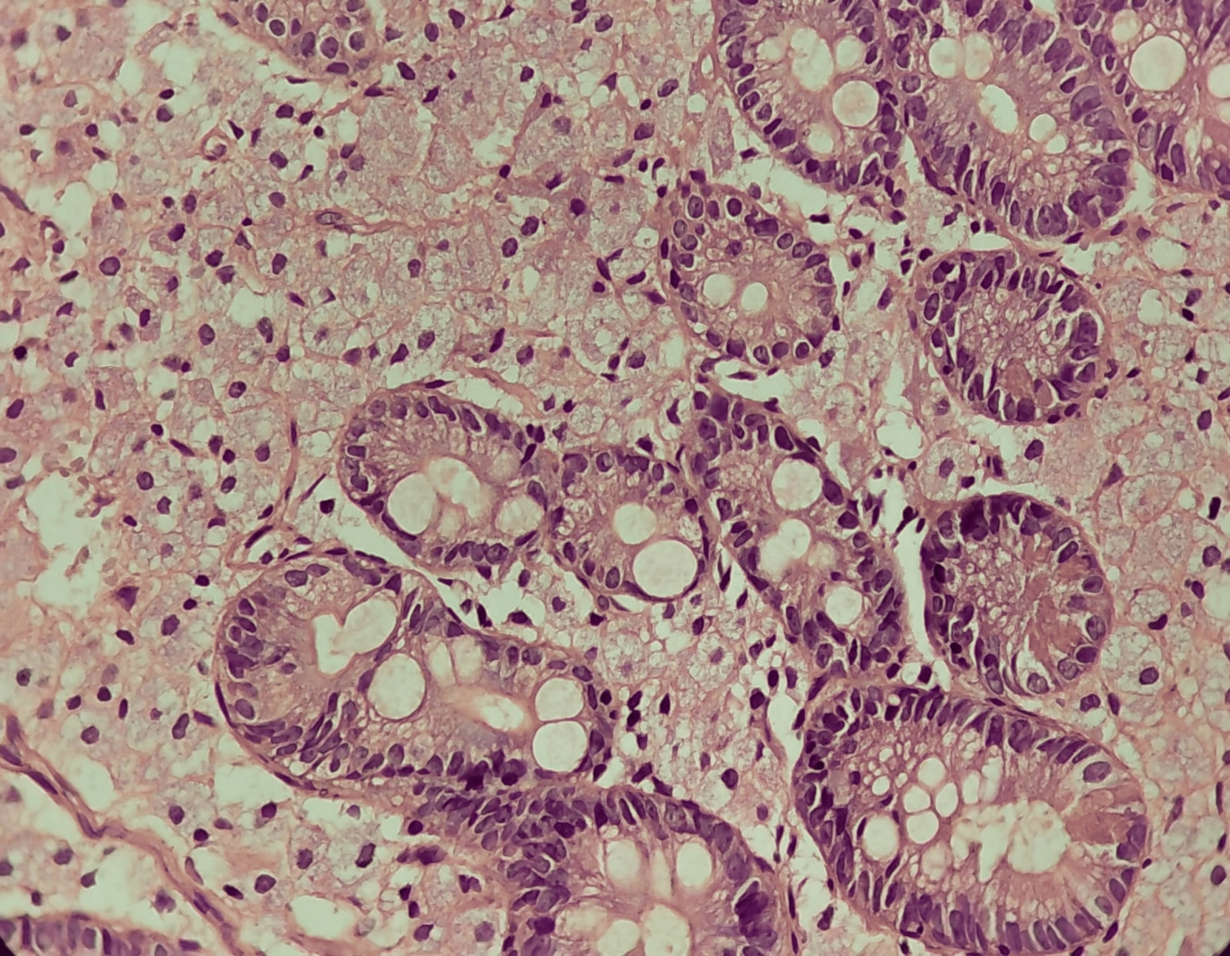
Hematoxylin and eosin stain: moderate histiocytic chronic duodenitis

**Figure 5:**
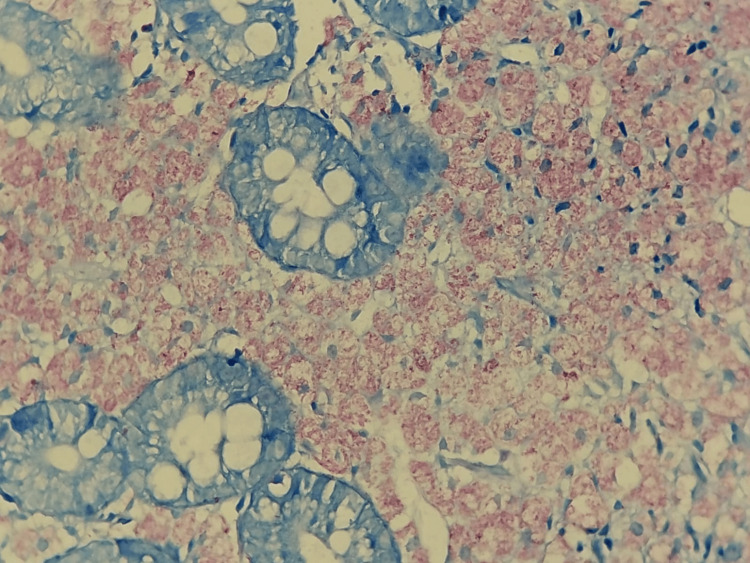
Ziehl-Neelsen stain: moderate histiocytic chronic duodenitis, associated with numerous intracellular mycobacteria. The search for acid-fast bacilli using Ziehl-Neelsen staining was positive (4+/4+), showing numerous intracellular mycobacteria

A new UGE performed in October 2021 (Figure 3) yielded histopathological findings of moderate chronic histiocytic duodenitis associated with numerous intracellular mycobacteria, with positive AFB staining (4+/4+) on Ziehl-Neelsen staining (Figures 4-5). This histological picture supported the clinical diagnosis of mycobacterial infection. The patient was then discharged on treatment for disseminated mycobacterial infection (MAC and MTBC) and herpes infection.

The patient discontinued treatment one month after discharge and was readmitted in April 2022, with persistent GIT complaints and a CD4 count of 28 cells/mm³. A new UGE (images unavailable) revealed hyperemic erosions measuring less than 5 mm located at the esophagogastric junction. The duodenal bulb and the beginning of the second portion of the duodenum showed diffuse moderate edema and hyperemia, with a whitish appearance over a velvety mucosal surface (differential diagnoses included lymphangiectasia or mycobacterial infection). Histopathological examination showed regenerative and inflammatory changes in the esophagogastric junction mucosa. In the duodenal portion, histological sections stained with hematoxylin and eosin, PAS, Grocott, and Ziehl-Neelsen revealed ulcerated duodenal mucosa with an inflammatory process characterized by a dense infiltrate of xanthomatous histiocytes dissociating the lamina propria, in addition to mild edema and vascular congestion. Histochemical staining was positive for AFB using the Ziehl-Neelsen method, with a large number of intracellular bacilli, and negative for fungi on PAS and Grocott stains. No pathognomonic morphological changes suggestive of viral infection or evidence of neoplasia were observed.

**Figure 6:**
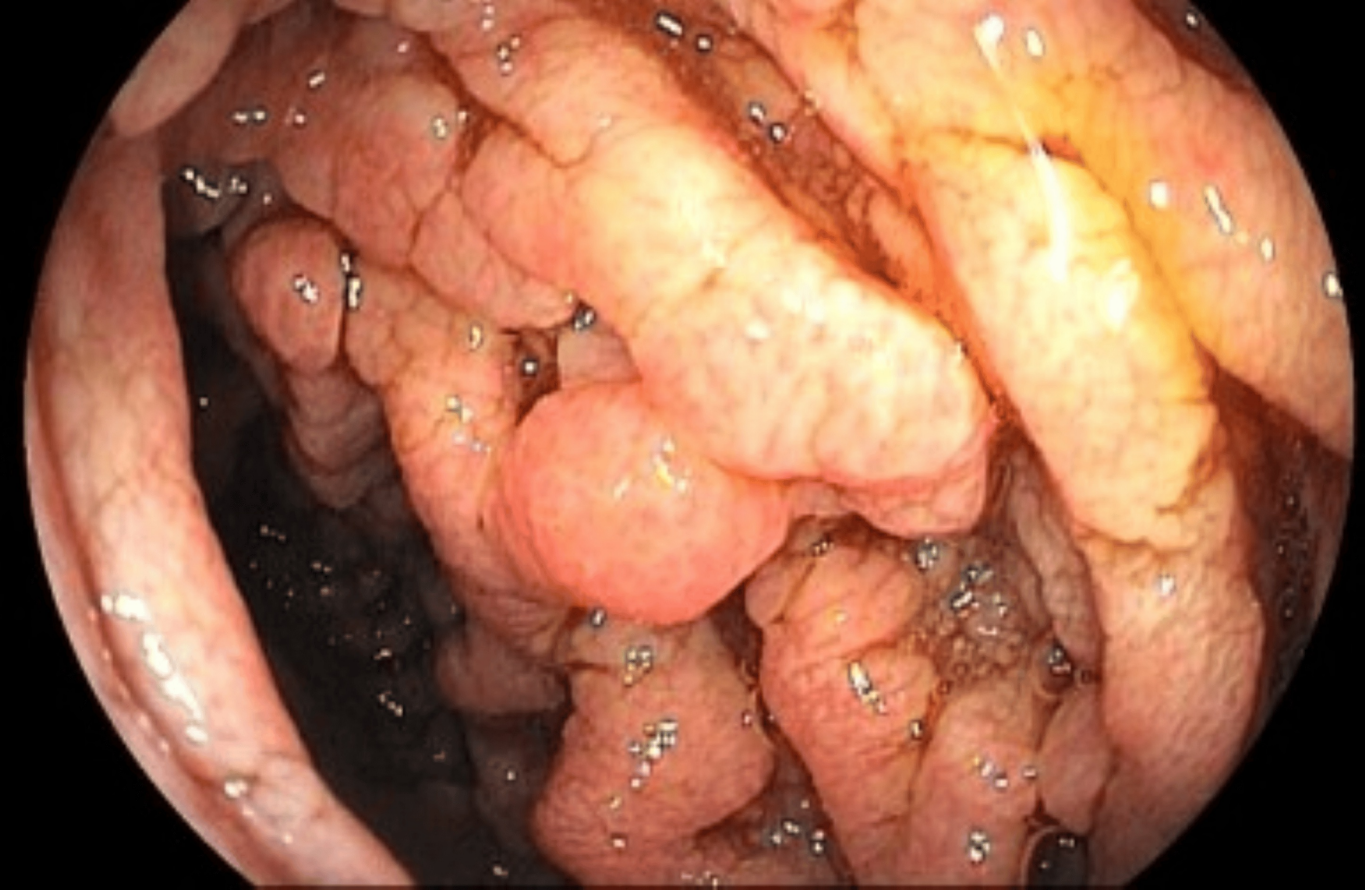
Duodenal bulb and second portion demonstrating pronounced diffuse hyperemia and edema, with irregular, granular mucosa interspersed with depressed, coarse areas (previous biopsy consistent with mycobacteriosis)

**Figure 7:**
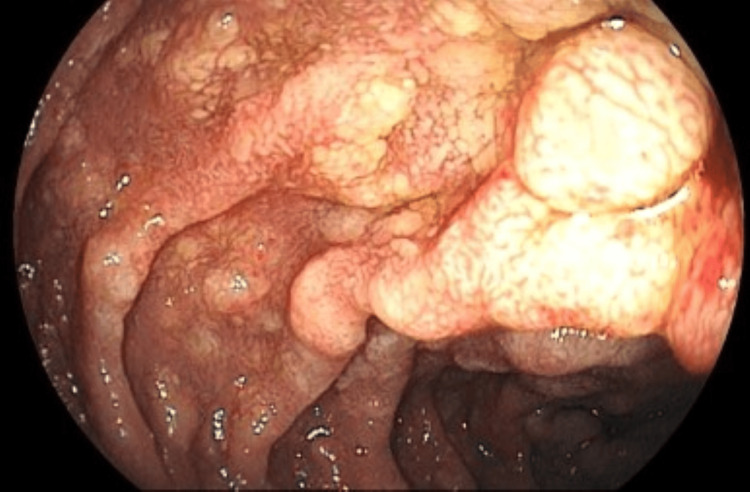
Duodenal bulb and second portion demonstrating pronounced diffuse hyperemia and edema, with irregular, granular mucosa interspersed with depressed, coarse areas (previous biopsy consistent with mycobacteriosis)

The patient was hospitalized again in December 2022, at which time the HIV-VL was 54,559 copies (log 4.74) and the CD4 count was 56 cells/mm³. Gastrointestinal complaints persisted, and a new UGE (Figures 6-7) showed the duodenal bulb and the beginning of the second portion of the duodenum with diffuse moderate hyperemia and edema and an irregular, granular, whitish mucosal surface interspersed with depressed and rough areas (again raising the differential diagnoses of lymphangiectasia or mycobacterial infection). Histopathological analysis once more demonstrated positive AFB staining on Ziehl-Neelsen, revealing numerous predominantly intracellular mycobacteria within the cytoplasm of histiocytes in the lamina propria of the duodenal mucosa.

**Figure 8:**
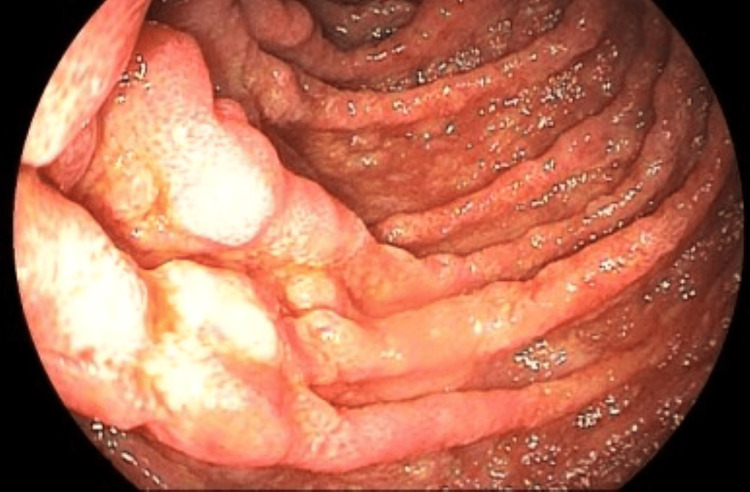
Duodenal bulb and second portion showing marked diffuse hyperemia and edema, with irregular, granular mucosa interspersed with depressed, coarse areas (previous biopsy consistent with mycobacteriosis)

In February 2023, the patient returned due to persistent gastrointestinal symptoms, at this time while receiving regular treatment for mycobacterial infection. A repeat UGE was performed (Figure 8), which continued to show diffuse hyperemia and edema of the duodenal bulb and second portion of the duodenum, more pronounced, with an irregular and granular mucosal surface interspersed with depressed and rough areas. Histopathological examination demonstrated chronic lymphohistiocytic duodenitis associated with the presence of numerous mycobacteria, with positive AFB staining on Ziehl-Neelsen, revealing numerous predominantly intracellular mycobacteria. In addition, parasitological investigations using routine stains and fungal studies using PAS and Grocott stains were negative, and no cytopathic changes indicative of viral infection were identified. Biopsy material was sent to the laboratory for AFB smear, Xpert MTB/RIF, and mycobacterial culture, all of which were negative or not detected.

Case 2

A 37-year-old patient, born and residing in São Paulo, PLWHA diagnosed in 2016, had been lost to follow-up for six years. At presentation, his HIV-VL was 222 copies, and his CD4 T-cell count was 57 cells/mm³. The patient had a previous history of tuberculosis diagnosed in 2016 (type not specified), confirmed by smear microscopy, for which he completed a six-month course of RHZE therapy and achieved cure. He presented to the ED with a one-month history of productive cough with greenish sputum, intermittent dyspnea, asthenia, myalgia, undocumented fever, poor appetite, and an 8-kg weight loss during this period.

**Figure 9:**
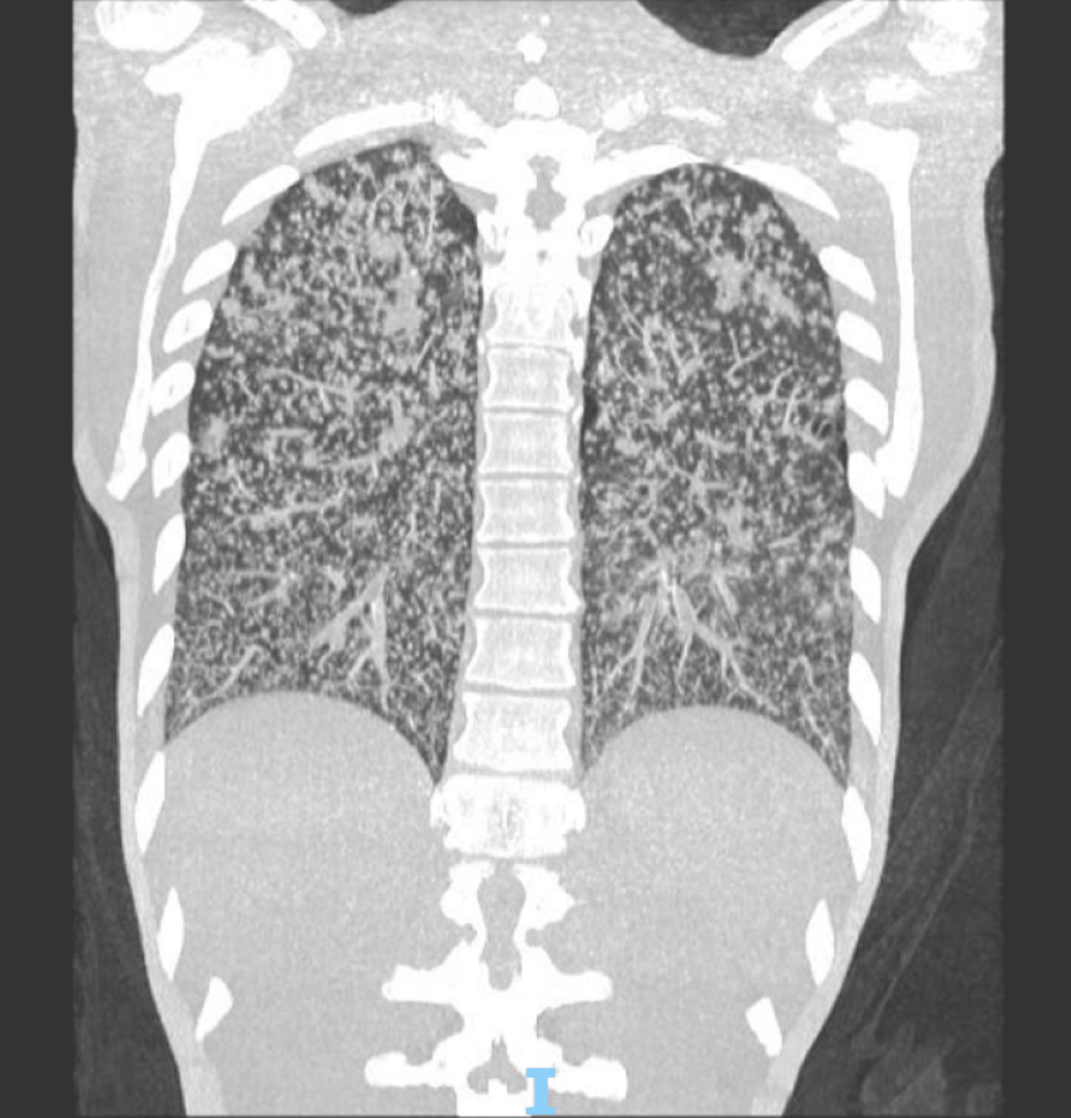
Chest CT scan demonstrating diffuse micronodular infiltrates, mild bilateral peribronchovascular consolidations, and diffuse thickening of the bronchial walls with an infectious-inflammatory pattern, findings consistent with miliary tuberculosis CT: computed tomography

One week prior, an external diagnosis of miliary tuberculosis had been established based on a positive sputum Xpert MTB/RIF and chest CT scan (Figure 9) showing diffuse micronodular infiltrates, mild scattered focal peribronchovascular consolidations bilaterally, and diffuse bronchial wall thickening with an infectious-inflammatory pattern, consistent with miliary tuberculosis, as well as enlarged mediastinal lymph nodes and cardiomegaly. At our institution, new tracheal secretion samples were collected, with positive AFB smear, detected Xpert MTB/RIF, and culture positive for the MTBC. The patient was admitted for treatment due to acute respiratory failure and transferred to the intensive care unit (ICU).

During hospitalization, pancytopenia was identified (hemoglobin 10.5 g/dL, leukocytes 3,900/mm³, platelets 75,000/mm³). An investigation for disseminated tuberculosis was conducted, and abdominal CT revealed hepatosplenomegaly associated with multiple small scattered hypodense lesions in the hepatic and splenic parenchyma (suggestive of microabscesses) and small-volume ascites, leading to the diagnosis of disseminated tuberculosis. Cerebrospinal fluid analysis showed negative or undetected results for AFB smear, Xpert MTB/RIF, and mycobacterial culture.

**Figure 10:**
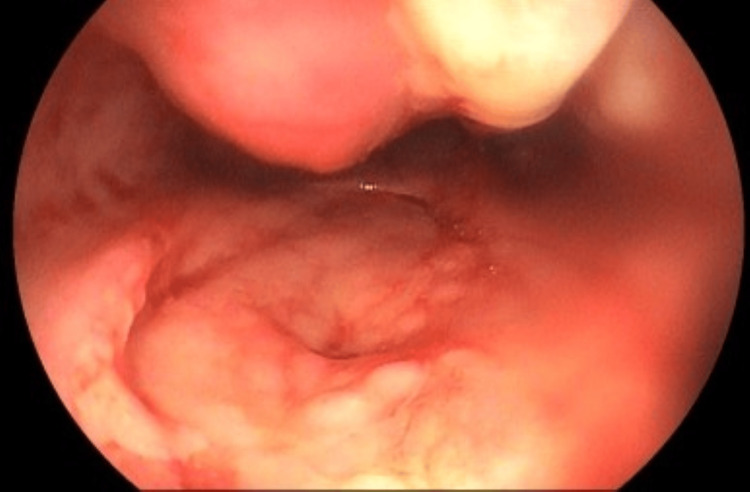
Multiple ulcerated esophageal lesions, some superficial and others deep, the largest of which is located on the posterior wall, 35 cm from the upper dental arch

**Figure 11:**
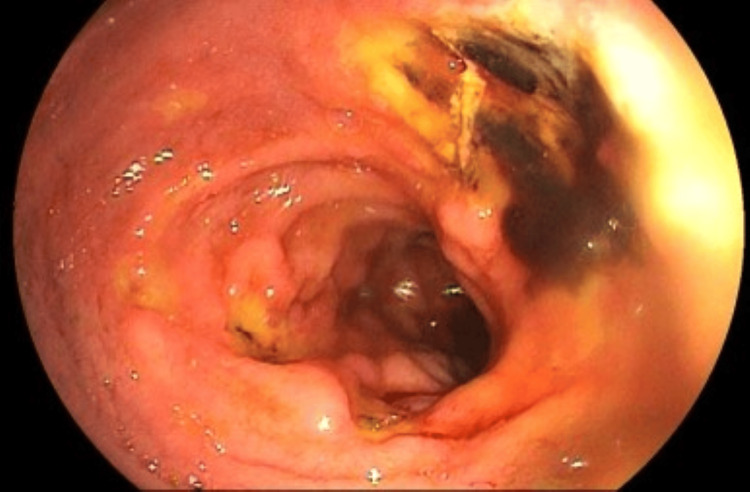
Duodenal bulb presenting with numerous oval-shaped ulcers of varying sizes, predominantly shallow, covered by a thick layer of yellowish fibrin. A notably deep ulcer is observed on the posterior wall of the mid-bulbar region, featuring a necrotic-appearing base with dense fibrin, hematin deposits, and loosely adherent blackened clots

The patient subsequently developed upper gastrointestinal bleeding (UGIB), with three episodes of melena, without hemodynamic instability. UGE was performed and revealed (Figure 10) multiple ulcerated lesions in the esophagus, the largest located on the posterior wall at 35 cm from the upper dental arch. In the duodenum (Figure 11), numerous shallow ulcers and one deep ulcer with a visible vessel stump, without active bleeding, were observed and treated with epinephrine injection. On the same day, the patient's antiretroviral therapy (ART) was restarted. After the visualization of ulcerated lesions on UGE, treatment for HSV infection was also initiated.

**Figure 12:**
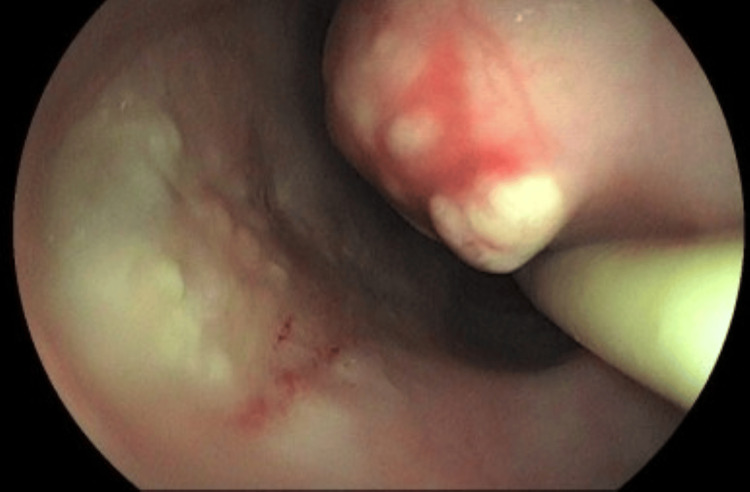
At 35 cm from the upper dental arch, a deep ulcerative lesion is observed on the posterior esophageal wall, characterized by a hemorrhagic base covered with fibrinous exudate and irregular margins. In the same region, on the anterior wall, there is a protruding lesion covered by mildly erythematous mucosa, with central fibrin deposition over a pulsatile area (possible vascular structure) partially occupying the esophageal lumen

**Figure 13:**
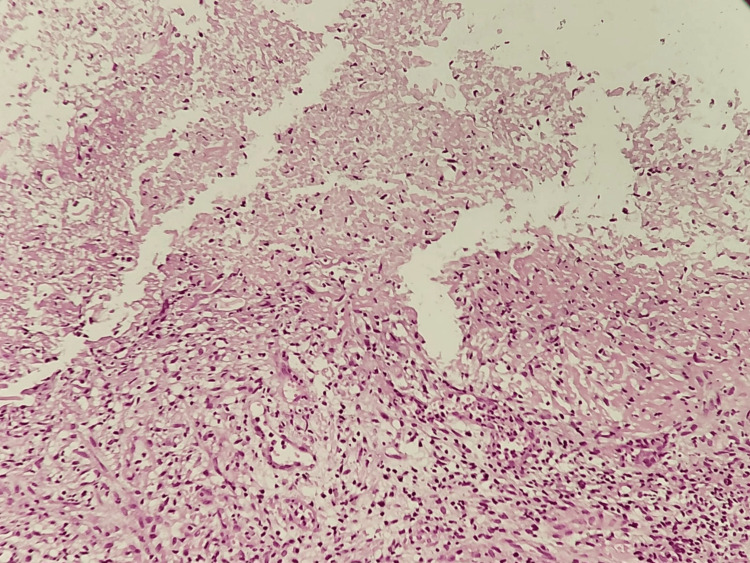
Hematoxylin and eosin stain: ulcerated esophageal mucosa with mildly thickened epithelium due to acanthosis and exocytosis

**Figure 14:**
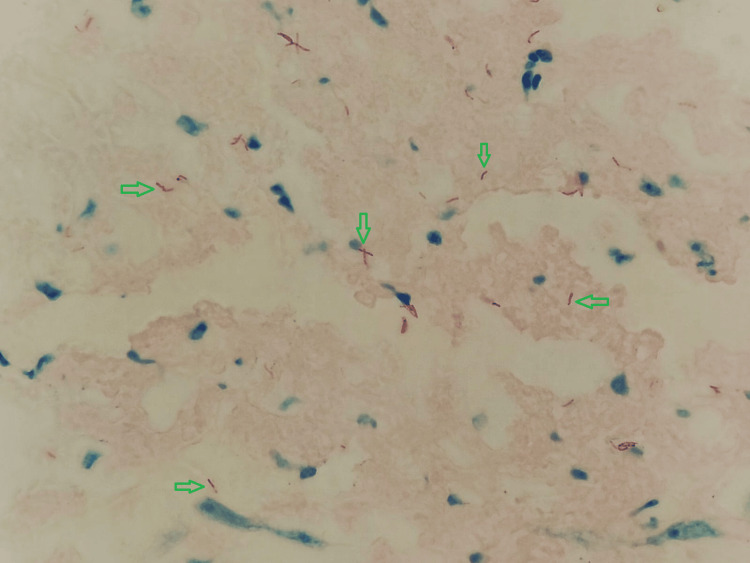
Ziehl-Neelsen stain: histochemical staining positive for acid-fast bacilli (green arrows) using the Ziehl-Neelsen method

Five days later, the patient developed a new episode of UGIB, and a repeat UGE was performed (Figure 12). This examination revealed the progression of the deep ulcerated lesion on the posterior esophageal wall (35 cm from the upper dental arch) to a lesion with a hemorrhagic base covered by fibrinous exudate and irregular margins. At the same level on the anterior wall, a bulging lesion (suspected to be extrinsic compression) covered by edematous mucosa with a finely granular appearance was observed, with a central protruding whitish area (suggestive of fibrin or caseous necrosis), without hematin or clots. A biopsy of the posterior wall lesion was performed, and histopathological analysis revealed ulcerated esophageal mucosa with mildly thickened epithelium due to acanthosis and exocytosis (Figure 13). In the deeper layers, a lymphocytic and histiocytic granulomatous reaction was observed amid necrosis. Histochemical staining was positive for AFB using the Ziehl-Neelsen method and negative for fungi on PAS and Grocott stains (Figure 14). In the duodenal bulb and the beginning of the second portion of the duodenum, numerous oval-shaped ulcers of varying sizes were observed, most of them shallow, covered by a thick yellowish fibrin layer. Histopathological examination of the duodenal ulcer revealed reactive duodenal mucosa with a lymphohistiocytic reaction in the lamina propria, in addition to edema and vascular congestion; however, histochemical staining was negative for AFB and fungi on Ziehl-Neelsen, PAS, and Grocott stains.

**Figure 15:**
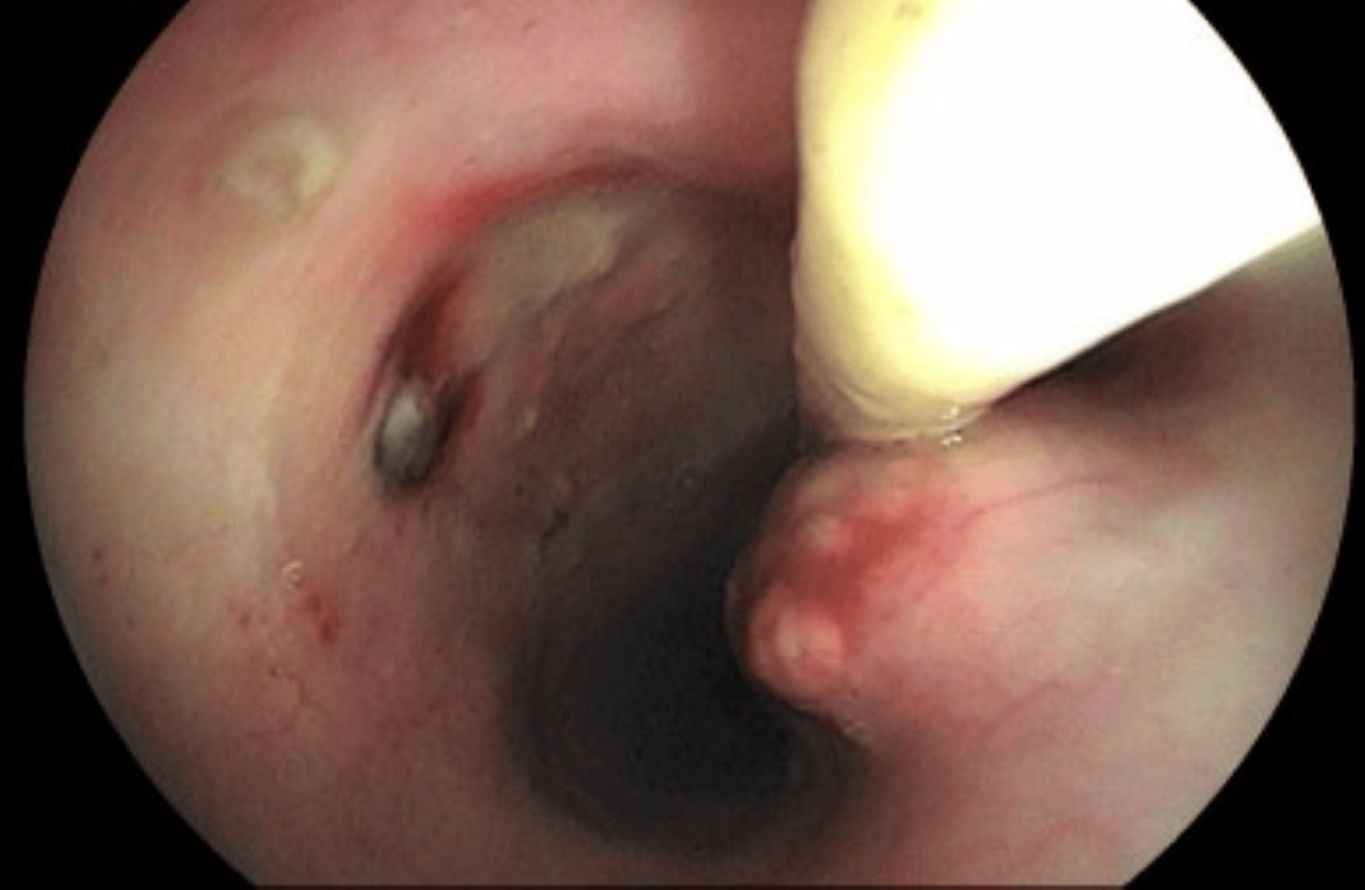
At 35 cm from the upper dental arch, on the posterior esophageal wall, a small ulcerated lesion is noted with a hemorrhagic base covered by fibrin and hematin (recent biopsy site). Adjacent to the lesion, a small diverticular outpouching is observed, possibly a traction diverticulum. On the anterior wall, in the same region, there is a bulging area, potentially of extrinsic origin, lined with edematous mucosa exhibiting a finely granular surface and a central protruding whitish area, suggestive of fibrin deposition or caseous necrosis, without evidence of hematin or clot formation

A follow-up UGE performed two days later (Figure 15) due to another episode of melena showed that the biopsied area had evolved into an ulcerated lesion with a hemorrhagic base covered by fibrin and hematin, with a small adjacent diverticular formation (suspected traction diverticulum). During hospitalization, the patient experienced gradual clinical deterioration and, despite the therapeutic measures implemented, progressed to candidemia, refractory septic shock, and death.

Case 3 

A 28-year-old patient, born in São Paulo, PLWHA diagnosed in 2018, had been lost to follow-up for approximately three months. At presentation, his HIV-VL was 53 copies (1.73 log), and his CD4 T-cell count was 48 cells/mm³. He had a diagnosis of pulmonary tuberculosis, with a sputum culture from 2021 positive for the MTBC, without mutations associated with resistance to rifampin or isoniazid. He discontinued treatment after three months of RHZE therapy and restarted it one week prior to hospital admission.

The patient presented to the ED with a 20-day history of poor appetite, nausea, vomiting, evening fever (39°C), abdominal pain, and diarrhea (approximately seven episodes per day), associated with weight loss.

**Figure 16:**
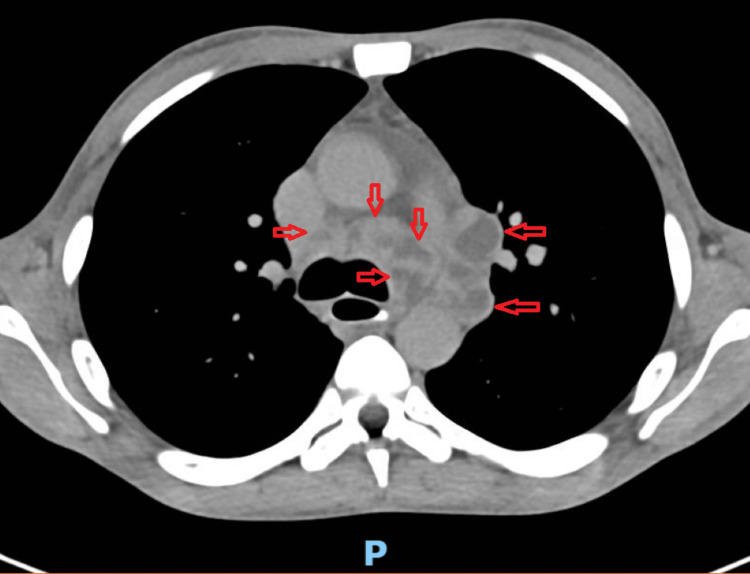
Presence of multiple oval-shaped images with lobulated and irregular contours, located in the mediastinum (red arrows), predominantly in the aortopulmonary window and the pre-vascular space, confluent, with peripheral enhancement and central liquid density (25-30 HU)

**Figure 17:**
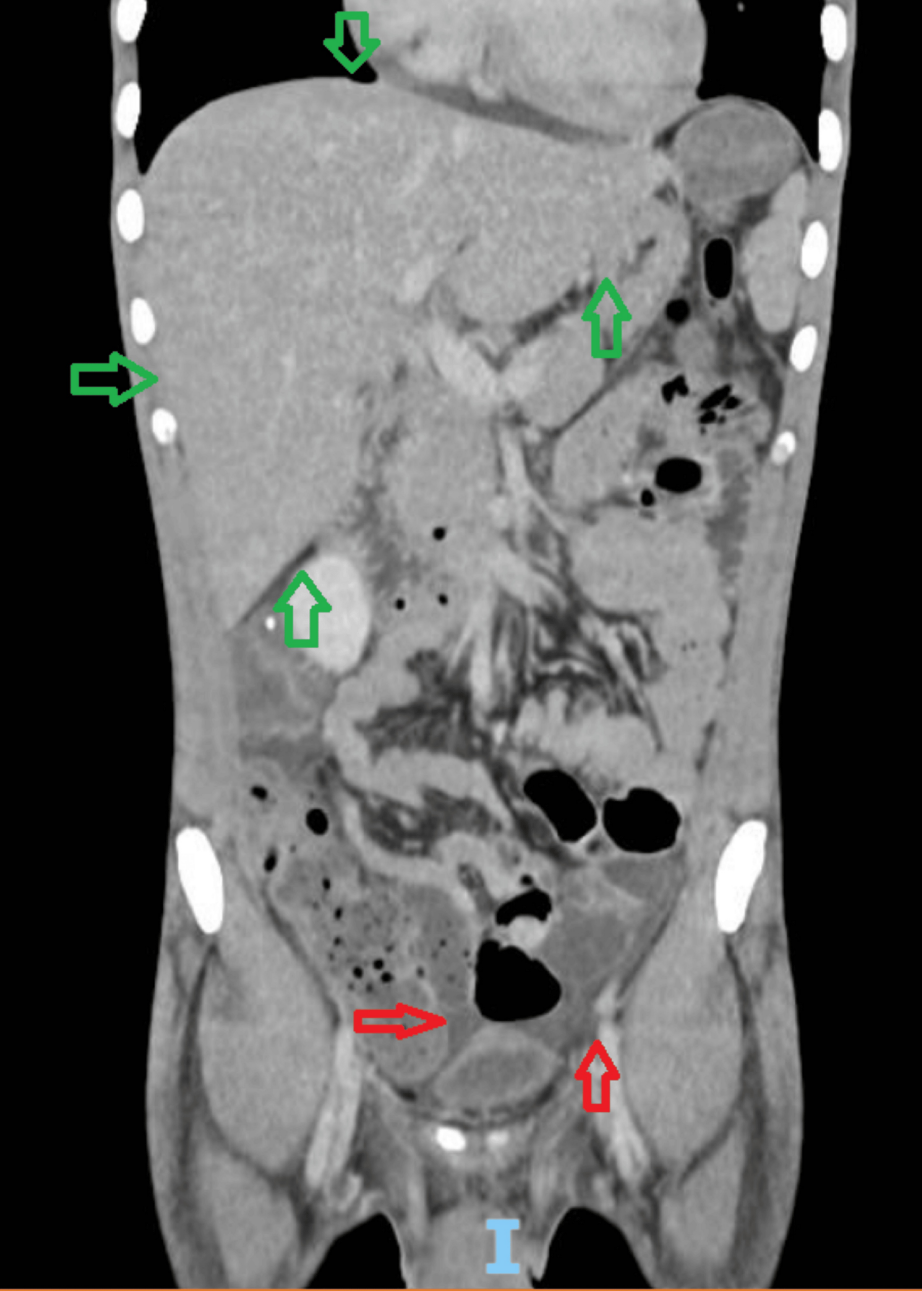
Axial contrast-enhanced abdominal CT scan demonstrating homogeneous hepatosplenomegaly (green arrows) and a small volume of ascites (red arrows) CT: computed tomography

At admission, disseminated tuberculosis (pulmonary and GIT involvement) was suspected based on clinical findings and chest CT (Figure 16), which showed subaortic and mediastinal lymphadenopathy, as well as a few scattered pulmonary micronodules. Abdominal CT (Figure 17) revealed homogeneous hepatosplenomegaly and small-volume ascites. Repeated sputum examinations performed at our institution showed negative AFB smear and undetected Xpert MTB/RIF; however, mycobacterial culture was positive for the MTBC, without mutations associated with resistance to rifampin or isoniazid.

Cerebrospinal fluid analysis with AFB smear, Xpert MTB/RIF, and mycobacterial culture yielded negative or not detected results. Stool investigations were performed and were all negative, including testing for *Clostridioides difficile *toxins A and B, screening for opportunistic pathogens (*Cryptosporidium *sp. and *Isospora belli*), and parasitological examinations (Ritchie method for helminths and protozoa; Lutz/Hoffman, Pons, and Janer methods for helminths and protozoa; and modified Rugai method).

On oral examination, whitish plaques were observed on the buccal mucosa, and antifungal therapy for candidiasis was initiated. A repeat stool workup was also entirely negative, including direct microscopy for amoebae, ELISA for *Cryptosporidium *sp., eosinophil and leukocyte testing, screening for opportunistic pathogens (*Cryptosporidium *sp. and *Isospora belli*), parasitological examinations (Ritchie, Lutz/Hoffman, Pons, and Janer, and modified Rugai methods), and fresh smear examination for larvae.

**Figure 18:**
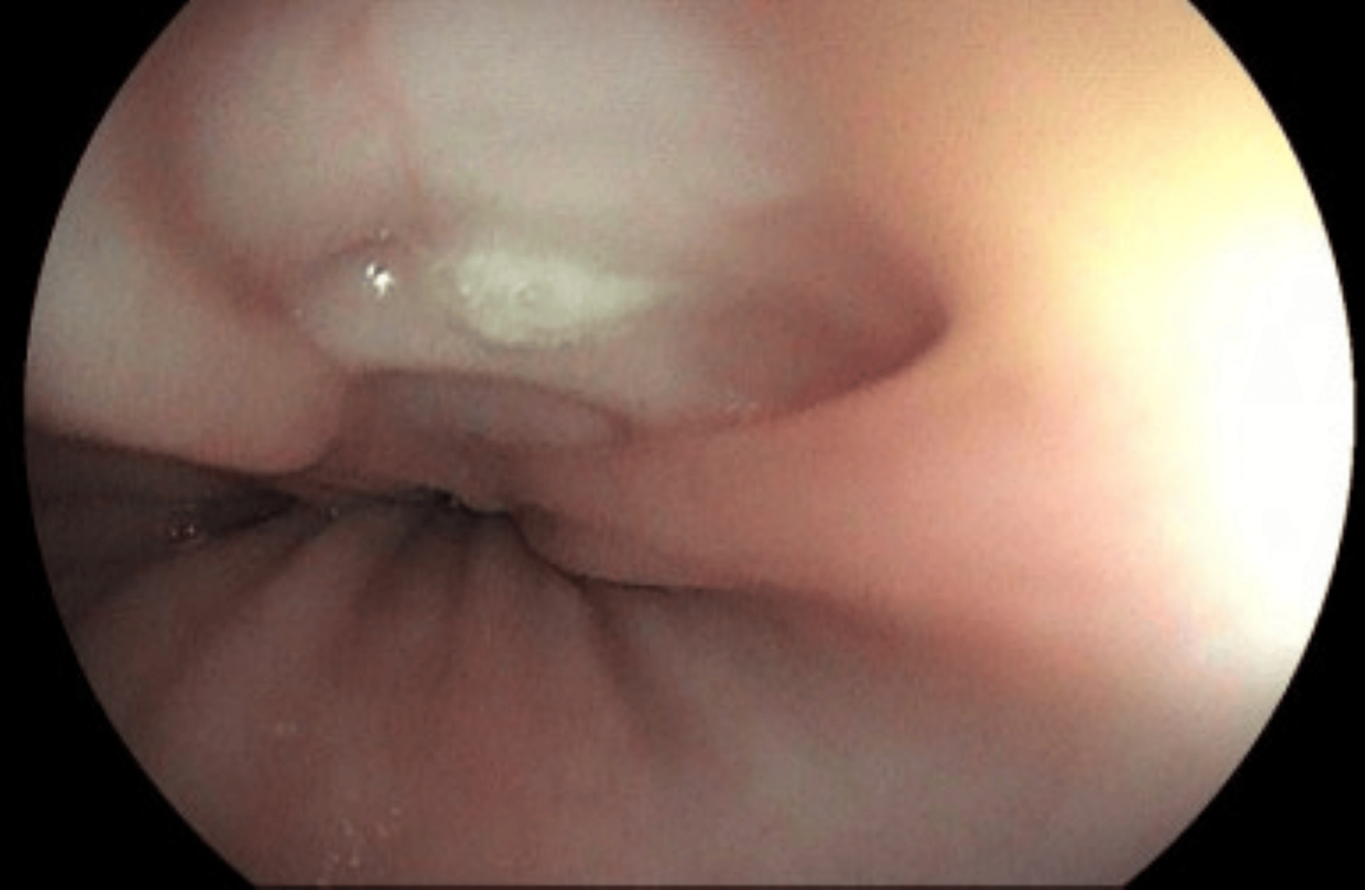
Oval-shaped ulcerated lesion located between 30 cm and 34 cm from the upper dental arch, appearing healed and re-epithelialized. However, a small fistulous orifice (~0.3 cm in diameter) is noted at the lesion's margin, with purulent discharge observed during the examination. Surrounding mucosa appears discolored

**Figure 19:**
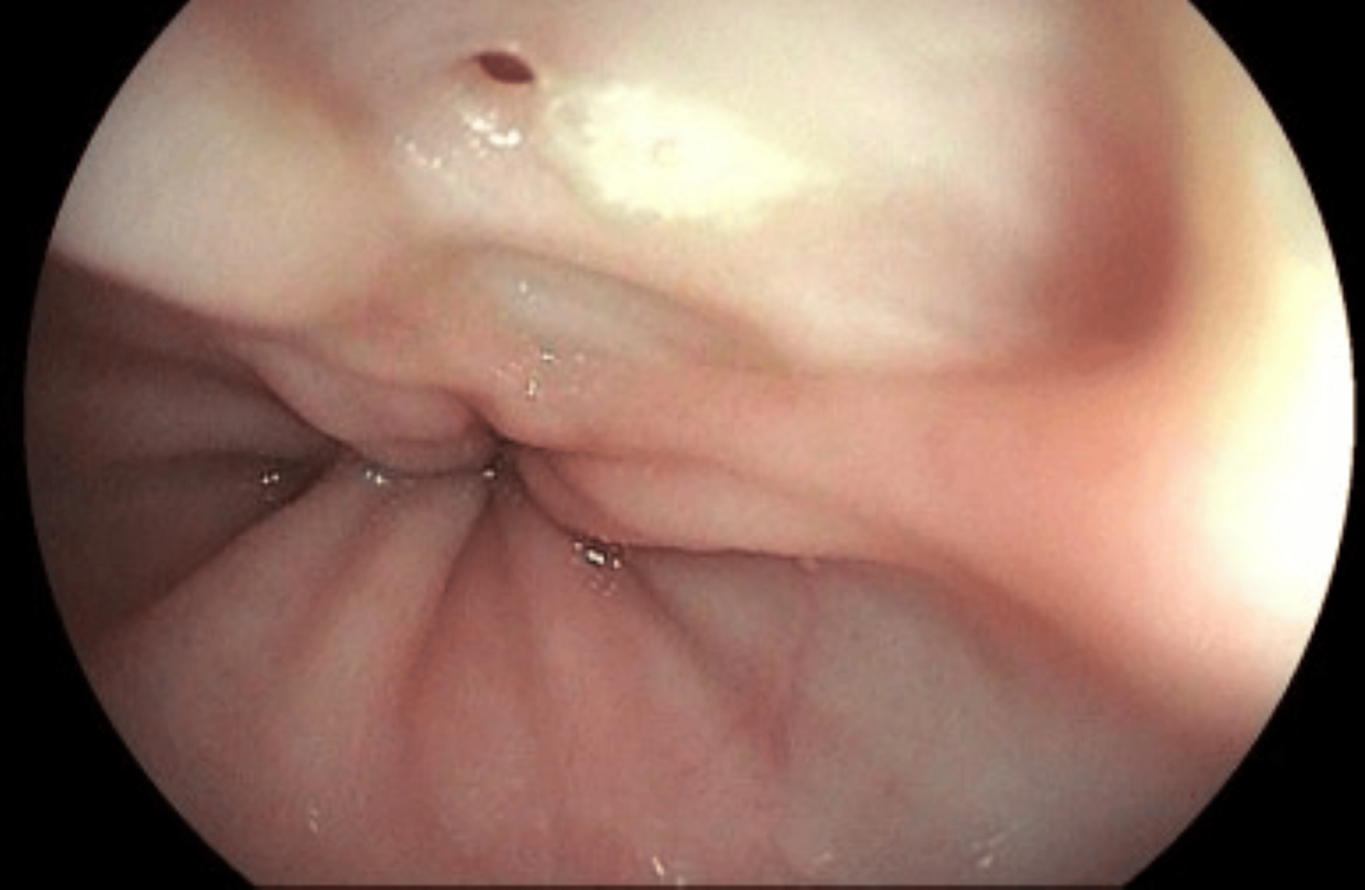
Oval-shaped ulcerated lesion located between 30 cm and 34 cm from the upper dental arch, appearing healed and re-epithelialized. However, a small fistulous orifice (~0.3 cm in diameter) is noted at the lesion's margin, with purulent discharge observed during the examination. Surrounding mucosa appears discolored

A UGE was performed for further investigation (Figures 18-19). In the esophagus, at 30-34 cm from the upper dental arch, on the anterolateral wall, an oval ulcerated lesion was observed, apparently healed and re-epithelialized, but with a small fistulous orifice (approximately 0.3 cm in diameter) at its margin, with purulent discharge, as well as pale esophageal mucosa. In the duodenum, markedly pale mucosa and mild diffuse edema were noted, without ulcers or scars. Biopsies were obtained from the esophageal lesion for CMV and mycobacterial investigation and from the duodenum for mycobacterial and *Cryptosporidium *testing. Histopathological examination of the esophagus revealed only squamous mucosa with moderate acanthosis and mild spongiosis, with negative AFB and fungal stains. Immunohistochemistry was negative for CMV and HSV type 1. In the duodenum, moderate chronic duodenitis was observed, with negative results for *Cryptosporidium*, AFB, and fungal stains.

**Figure 20:**
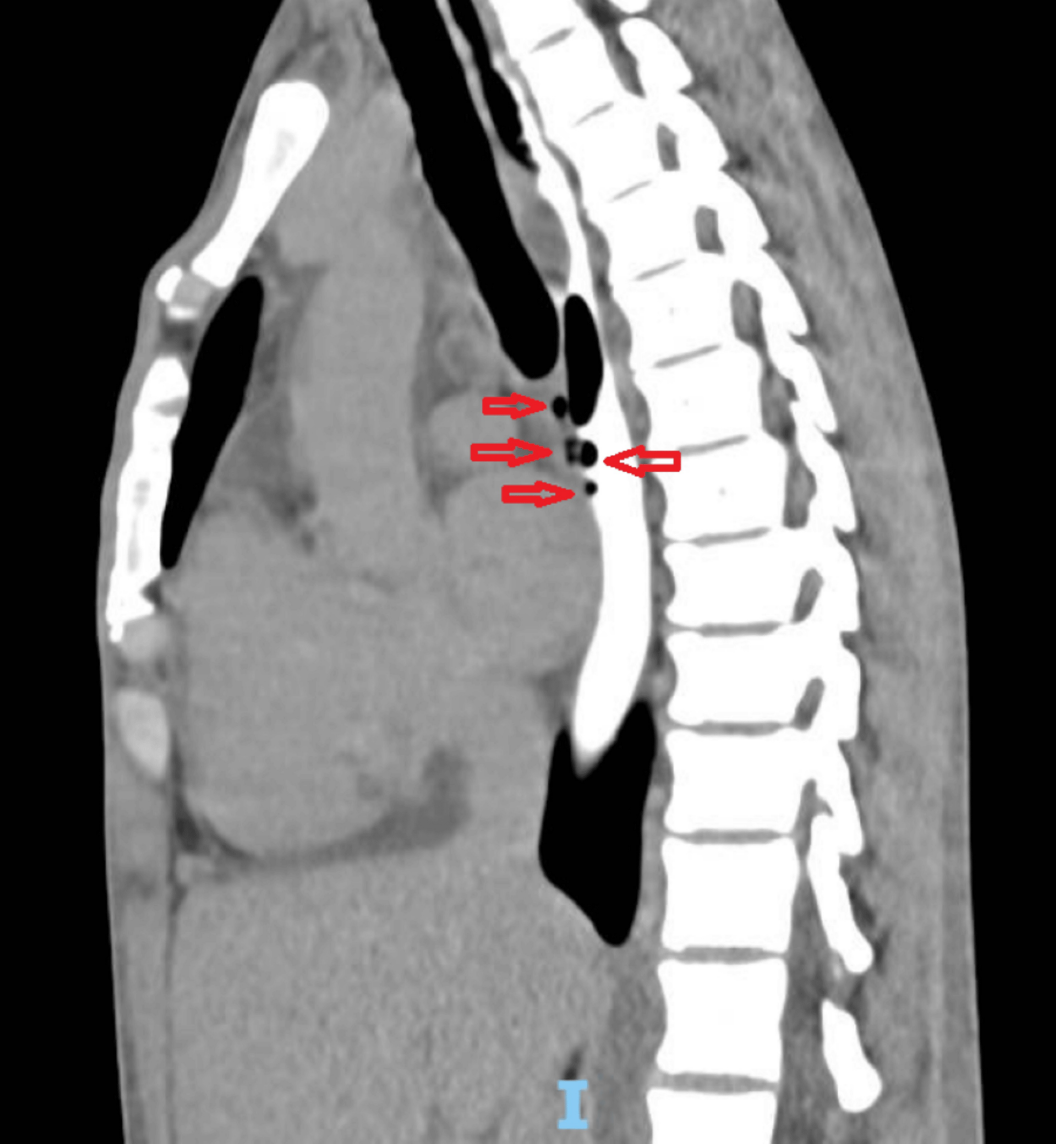
Chest CT scan with double contrast (intravenous and oral) demonstrating, at the middle third of the esophagus, the presence of gas bubbles projecting from the esophageal lumen toward the anterior wall (red arrows), suggestive of a possible fistulous tract following oral contrast administration CT: computed tomography

A new chest CT scan with dual contrast (intravenous and oral) (Figure 20) revealed multiple oval lesions with lobulated and irregular contours in the mediastinum, with peripheral enhancement and central fluid density, greater in number and size compared to the previous examination. The esophagus was also distended along its entire length, and after the administration of oral contrast, gas bubbles were observed at the level of the mid-esophagus apparently projecting from the esophageal lumen toward the anterior wall, raising suspicion of a fistulous tract. Mediastinoscopy was then performed to evaluate the suspected fistulous tract and to obtain biopsies of mediastinal lymph nodes, which appeared necrotic and with purulent secretion. The material was sent for histopathological analysis, which revealed granulomatous lymphadenitis due to mycobacteria, with AFB staining showing a moderate number of bacilli within the inflammatory process. In addition, lymph node samples were AFB-positive with detected Xpert MTB/RIF, and mediastinal secretion was also AFB-positive, although mycobacterial culture was negative. The patient was discharged for continued treatment and follow-up at his referring institution.

## Discussion

Many pathogens can cause opportunistic infections of the GIT, with mycobacterial infections being one of the main possibilities, especially those caused by the MTBC and NTM [2,6,16,17]. The clinical presentation and diagnostic workup can be similar for both etiologies [16,17]. Maintaining a high level of clinical suspicion is essential, as these infections may present along a broad spectrum, ranging from asymptomatic forms and nonspecific manifestations to classic presentations with fever, cough, and weight loss [5,17,18]. Furthermore, in GIT involvement, both clinical manifestations and endoscopic findings are highly nonspecific, with a wide range of possible presentations [1,6].

Tuberculosis (MTBC)

The MTBC comprises a group of closely related species, including *M. tuberculosis *sensu stricto, *M. africanum*, *M. bovis*, *M. orygis*, *M. microti*, and *M. caprae*, among others [5,13]. According to World Health Organization (WHO) data, tuberculosis affects approximately 10 million people annually and causes around one million deaths, making it the leading cause of death from a single infectious agent worldwide [19]. Of these cases, in 2024, 5.8% occurred in PLWHA, representing a decrease from 6.1% in 2023 [19].

Anyone can be infected with MTBC; however, the risk of progression to active disease is higher among immunosuppressed individuals [17,18]. Table 1 summarizes the characteristics of the sample in this case series, highlighting the representation of a subgroup with low CD4 T-cell counts, below 100 cells/mm³. This mycobacterial infection can affect any organ in the body, with extrapulmonary tuberculosis accounting for approximately 12-15% of patients with active tuberculosis globally, although this proportion may vary by region [5,6,20]. Gastrointestinal tuberculosis is rare, representing approximately 1-3% of all tuberculosis cases, and is considered only the sixth most common form of extrapulmonary tuberculosis (EPTB), accounting for 10-16% of EPTB cases, with lymph node involvement being the most common [5,6,20].

**Table 1:** Characteristics of the patients in the case series HIV: human immunodeficiency virus; HIV-VL: human immunodeficiency virus viral load; CD4: CD4 T-cell count

Characteristics	Case 1	Case 2	Case 3
Age (years)	27	37	28
Duration of HIV infection (years)	25	7	5
HIV-VL (copies)	279.831	222	53
CD4 (cc/mm³)	16	57	48

GIT involvement may be primary or associated with pulmonary disease and can affect any part of the tract, viscera, or peritoneum [5]. The most commonly affected sites are the peritoneum and intestines, and the esophageal involvement is one of the rarest forms of gastrointestinal tuberculosis, accounting for approximately 0.2-0.3% to 2.8% of cases, despite the anatomical proximity to the lungs [5,6,20]. Even so, the most widely accepted mechanism of esophageal involvement is contiguous spread from adjacent mediastinal structures, particularly lymph nodes in the mid-esophagus near the carina [5]. PLWHA are at higher risk for peritoneal tuberculosis [5].

In most cases, infection at this site occurs as a secondary process, through either hematogenous or lymphatic dissemination, by swallowing infected respiratory secretions or ingesting contaminated materials (usually unpasteurized milk), by contiguous spread from infected mediastinal lymph nodes, or, in the case of intestinal involvement, through the excretion of contaminated bile from an infected liver, being rare the primary infection cases [5,6,18]. When caused by MTBC, constitutional symptoms such as fever, night sweats, anorexia, and weight loss are usually present, in addition to nausea, vomiting, abdominal pain, diarrhea, or constipation [5,6]. The clinical presentation may vary depending on the segment of the GIT involved, and anatomical differentiation based solely on clinical findings is difficult [5]. Nonspecific abdominal pain may be present in 80-90% of cases, as well as abdominal distension secondary to ascites, typically in cases of peritoneal involvement [5,20]. When the esophagus is affected, odynophagia, dysphagia, particularly prominent, and chest pain may also occur [5,20]. On physical examination, ascites, hepatomegaly, and splenomegaly are noteworthy [5], findings present in all three cases in our series. In PLWHA, clinical manifestations may vary, with ascites, para-aortic lymphadenopathy, hepatosplenomegaly, and mesenteric masses being common [5]. Table 2 summarizes the clinical features observed in the patients included in this case series.

**Table 2:** Symptoms presented by the patients X: present; -: absent

Symptoms	Case 1	Case 2	Case 3
Cough	X	X	-
Dyspnea	X	X	-
Fever	X	X	X
Weight loss	X	X	X
Asthenia	-	X	-
Myalgia	-	X	-
Poor appetite	X	X	X
Dysphagia	X	-	-
Postprandial fullness	X	-	-
Nausea	-	-	X
Vomiting	-	-	X
Abdominal pain	-	-	X
Diarrhea	X	-	X

NTM

NTM are those that do not belong to the *M. tuberculosis *complex or the *M. leprae *complex and, according to some classifications, also exclude *M. ulcerans*; they comprise more than 190 species [12,16,21,22]. They are classified as slow-growing or rapidly growing mycobacteria, depending on whether growth occurs in more or less than seven days, respectively [12,21,22]. Among the rapidly growing mycobacteria are the MABC, *M. fortuitum *complex, and *M. chelonae *[12]. Among the slow-growing species, *M. kansasii*, *M. marinum*, *M. gordonae*, *M. ulcerans*, and *M. xenopi *are noteworthy [12,16].

These organisms are environmental pathogens and can be found in soil, water, dust, and plants [12,21,22]. They are generally of low pathogenicity to humans but can cause opportunistic infections, with a currently increasing incidence [12,21,22].

The primary system affected by NTM infections is the pulmonary system; however, lymph nodes, skin and soft tissues, bones, joints, the central nervous system, the GIT, or disseminated disease, particularly in immunocompromised individuals, may also be involved [12,21,22]. There is an association between slow-growing NTM and a higher frequency of pulmonary and lymph node infections, whereas rapidly growing NTM are more commonly associated with skin, bone, and joint infections [22].

In disseminated NTM infections, the main routes of infection involve environmental exposure through the respiratory tract and GIT, with person-to-person transmission being virtually excluded [22]. After exposure, GIT colonization may occur through adhesion to the intestinal mucosa, penetration of the lamina propria, and phagocytosis by macrophages [1,3,23]. From this site, organisms may penetrate the submucosa, reach the lymphatic system, and cause hematogenous dissemination, potentially involving the bone marrow and spleen and leading to anemia [1,3,23]. However, initial presentation in organs other than the lungs is rare in the absence of pulmonary involvement [1].

In NTM infections, the most common clinical presentation is pulmonary, particularly in individuals with structural airway abnormalities, and may be associated with pulmonary or systemic symptoms [16]. GIT infection caused by NTM resembles intestinal tuberculosis as well as inflammatory bowel disease [21]. The clinical picture is characterized by high fever, diarrhea, weight loss, abdominal pain, sweating, anemia, hepatomegaly, and splenomegaly [22].

MAC

MAC is a subgroup of NTM composed of several species, including *M. avium*, *M. intracellulare*, and *M. chimaera *[12,16]. With the advancement of molecular diagnostic techniques, additional species have been described as belonging to the MAC group; however, their clinical significance has not yet been clearly established [11]. MAC is the most frequent cause of NTM infection, particularly involving pulmonary and disseminated disease, and is responsible for significant morbidity and mortality worldwide, with increasing incidence due to the growing population of immunocompromised patients [1,3,5,11,12].

In PLWHA, epidemiological studies show variable prevalence rates, ranging from 10% to 20-40%, and MAC causes more severe infections in this group, usually presenting in the disseminated form and often involving the GIT [1,5,24]. Clinically, MAC infection may present with fever, weight loss, and malabsorption [5].

Diagnosis

In the investigation of GIT mycobacterial infections, radiographic studies, particularly CT, and endoscopic procedures, such as UGE and colonoscopy, are useful tools [5,6,20]. CT scans are especially helpful for detecting lymphadenopathy, with hilar and mediastinal involvement being quite common [6,20]. Other possible findings include asymmetric thickening of the intestinal wall or, in hypertrophic forms, mass-like lesions [5]. The importance of CT imaging was highlighted in the case report by Morare et al., in which esophageal tuberculosis was diagnosed secondary to invasion of the esophageal wall via mediastinal lymphadenopathy [6], similar to what was observed in our third case. Table 3 summarizes the laboratory and imaging findings of our cases. Contrast swallow studies may also be performed to evaluate mucosal ulcerations, luminal narrowing, strictures or fistulas, as well as ileocecal irritability or hypermotility [5,6,20]. However, the clinical, radiological, and endoscopic characteristics of gastrointestinal mycobacterial infection are not yet fully defined [6,20].

**Table 3:** Laboratory and imaging changes X: present; -: absent

Findings	Case 1	Case 2	Case 3
Anemia	X	X	-
Leukopenia	X	X	-
Thrombocytopenia	X	X	-
Ascites	Small volume	Small volume	Small volume
Hepatomegaly	-	X	X
Splenomegaly	X	X	X
Hepatic infarctions	-	-	-
Splenic infarctions	X	-	-
Hepatic hypodensity	-	X	-
Splenic hypodensity	-	X	-
Cardiomegaly	X	X	-
Lymphadenopathies	Retroperitoneal, mesenteric, and iliac	Mediastinal	Mediastinal and subaortic
Lung changes	Inflammatory bronchopathy	Inflammatory bronchopathy and diffuse micronodular infiltrate	Rare scattered pulmonary micronodules

Although UGE often reveals variable and nonspecific findings, it remains an important diagnostic tool in individuals with gastrointestinal symptoms and suspected HIV coinfection with disseminated mycobacterial disease [3,5,6,23]. The most common endoscopic findings include granulomas; elevated yellowish-white plaques and nodules; mucosal irregularity; edema; erosions and usually shallow ulcers; friability and/or decreased mucosal vascular pattern; clustered lesions; or extrinsic compressions in the esophagus [3,5]. Fistulas are less frequent but may also be observed in cases of gastrointestinal tuberculosis, particularly with esophageal involvement [6]. In MAC infection, yellow mucosal nodules have been described on duodenal endoscopy [5]. In esophageal tuberculosis, deep biopsies from the lesion margin are recommended due to the submucosal location of the disease [20]. In addition, obtaining multiple biopsy samples may increase diagnostic accuracy [5].

In our case series involving PLWHA, multiple and varied endoscopic presentations were also observed. In the esophagus, patterns included shallow and deep ulcerated lesions, a fistulous orifice with purulent discharge, bulging lesions covered by edematous mucosa with a finely granulomatous appearance, and pale mucosa. In the duodenum, findings included irregular and granular mucosa; markedly pale/whitish or velvety mucosa; diffuse hyperemia; diffuse edema; depressed and rough areas; multiple shallow or deep ulcers; and diverticular formation. Table 4 summarizes the endoscopic features of the lesions and the diagnoses of GIT mycobacterial infections in our case series.

**Table 4:** Endoscopic aspects of lesions and diagnosis of GIT mycobacterial infections UGE: upper gastrointestinal endoscopy; AP: anatomopathological; GIT: gastrointestinal tract; TS: tracheal secretion; AFB: acid-fast bacilli; Xpert MTB/RIF: rapid molecular test for tuberculosis; Culture: culture for mycobacteria; BAL: bronchoalveolar lavage; UDA: upper dental arch; CSF: cerebrospinal fluid; IHC: immunohistochemistry; -: not performed/unavailable

Diagnosis	Case 1	Case 2	Case 3
UGE 1	Duodenum with mucosa of irregular and granular appearance, whitish	Esophagus: multiple ulcerated lesions, the largest located on the posterior wall, 35 cm from the UDA. Duodenum: numerous shallow ulcers and one quite deep ulcer with a vascular stump, without active bleeding	Esophagus: 30-34 cm from the UDA, on the anterolateral wall, oval ulcerated lesion, apparently healed and re-epithelialized, but with a small fistulous opening (about 0.3 cm in diameter) on its margin, with purulent discharge, as well as discolored mucosa. Duodenum: mucosa is quite discolored with mild diffuse edema, without ulcers or scars
AP 1	Moderate chronic histiocytic duodenitis, associated with numerous intracellular mycobacteria, with positive AFB smear (4+/4+)	-	Esophagus: squamous mucosa with moderate acanthosis and mild spongiosis, negative AFB smear. IHC negative for CMV and HSV type 1. Duodenum: moderate chronic duodenitis, negative AFB smear
UGE 2	Bulb and beginning of the second portion of the duodenum with moderate diffuse edema and hyperemia, with a whitish appearance on the mucosa with a velvety aspect (suspected lymphangiectasia or mycobacteriosis)	Esophagus: progression of a deep ulcerated lesion on the posterior wall of the esophagus (35 cm from the UDA) to a lesion with a raw base covered by fibrinous exudate and irregular edges. In the same location, on the anterior wall, a bulge (extrinsic?) covered by edematous mucosa with a finely granular appearance, with a central protruding whitish area (fibrin? caseous necrosis?), without hematin or clots. Duodenum: bulb and beginning of the second portion, presence of numerous oval ulcers, of varying sizes	-
AP 2	Duodenal mucosa with ulceration and dense infiltrate of foamy histiocytes dissociating the lamina propria, mild edema, and vascular congestion; AFB smear positive with a large number of intracellular bacilli	Esophagus: lesion of the posterior wall with ulcerated esophageal mucosa, with epithelium slightly thickened due to acanthosis, with exocytosis. In depth, there is a lymphohistiocytic reaction of granulomatous pattern amid necrosis, with positive AFB. Duodenum: reactive mucosa, showing lymphohistiocytic reaction in the lamina propria, along with edema and vascular congestion, but negative AFB smear	-
UGE 3	Bulb and second portion of the duodenum with diffuse hyperemia and edema, pronounced, with mucosa appearing irregular and granular, interspersed with depressed and rough areas	Esophagus: biopsied area on the posterior wall evolved into an ulcerated lesion with a raw base covered by fibrin and hematin, with a small adjacent diverticular formation (hypothesis: traction diverticulum)	-
AP 3	Chronic lymphohistiocytic duodenitis associated with the presence of numerous mycobacteria, predominantly intracellular, with AFB smear positive by Ziehl-Neelsen staining	-	-
AP of GIT: AFB smear	Positive	Positive	Negative
AP of GIT: Xpert MTB/RIF	-	-	-
AP of GIT: culture	-	-	-
Blood culture	Negative	-	-
Sputum/TS: AFB smear	Negative	Positive	Negative
Sputum/TS: Xpert MTB/RIF	Not detected	Detected	Not detected
Sputum/TS: culture	Negative	Positive for MTBC	Positive for MTBC
BAL: AFB smear	Negative	-	-
BAL: Xpert MTB/RIF	Not detected	-	-
BAL: culture	-	-	-
Gastric lavage: AFB smear	Negative	-	-
Gastric lavage: Xpert MTB/RIF	-	-	-
Gastric lavage: culture	Negative	-	-
Lymph node: AFB smear	-	-	Positive
Lymph node: Xpert MTB/RIF	-	-	Detected
Lymph node: culture	-	-	-
Other sites: AFB smear	-	CSF: negative	Mediastinal secretion: positive
Other sites: Xpert MTB/RIF	-	CSF: not detected	-
Other sites: culture	Myelogram: negative	CSF: negative	Mediastinal secretion: negative

Diagnostic tools also include tests to detect mycobacteria, such as AFB smear, MTBC-specific PCR (Xpert MTB/RIF), and culture, which can be performed on various specimens, including sputum, secretions, blood, and biopsy samples [1,5,6,20,25]. One of the most important diagnostic tools is biopsy, with histological evaluation demonstrating features suggestive of granulomas, with or without caseous necrosis [1,6,20]. However, the presence of granulomas is not pathognomonic [20]. Definitive microbiological diagnosis is established by the gold standard methods: culture and/or molecular testing [25].

Histological descriptions of cases that later yielded positive mycobacterial tests often show the presence of histiocytes and giant cells [1,20]. Classic granulomas are present in only approximately 50% of cases [20]. In advanced immunosuppression among PLWHA, due to a poor inflammatory response, granuloma formation is uncommon, and inflammation without granuloma formation may occur, facilitating dissemination [5,26]. In addition, AFB staining is frequently positive, with a high bacillary load [5].

Unfortunately, the sensitivity and specificity of AFB smear, Xpert MTB/RIF, and culture for gastrointestinal tuberculosis are low [5,20]. Furthermore, gastrointestinal and peritoneal tuberculosis are often paucibacillary, which reduces the likelihood of positive results in noninvasive samples such as lavage fluids [5]. PCR sensitivity ranges from 74% to 100% in smear-positive samples [20]. In biopsy specimens, AFB smear and culture have sensitivities below 50%, and the sensitivity of endoscopic biopsies is even lower, at approximately 22% [20]. This finding is supported by Eraksoy, who reported an AFB sensitivity of 20.5% in intestinal mucosa, compared with a PCR sensitivity of 64.1% [5].

Another diagnostic tool is urinary lipoarabinomannan (LAM) detection [26]. This is a glycolipid component of the mycobacterial cell wall and may be positive in both MTBC and NTM infections [26]. Its sensitivity ranges from 29% in outpatients to 52% in hospitalized patients [26]. According to the WHO, this test is indicated for PLWHA who have signs or symptoms of tuberculosis, are severely ill, hospitalized in stage 3 or 4 disease, or have CD4 T-cell counts below 200 cells/mm³ for hospitalized patients or below 100 cells/mm³ for outpatients [26,27].

It is also possible, particularly in PLWHA with low CD4 counts, for all microbiological tests to be negative, with the diagnosis being established based on clinical and radiological criteria [26].

The tuberculin skin test (TST) and interferon-gamma release assays (IGRAs) are not useful for diagnosis, as they do not indicate active disease and may yield negative results in PLWHA with active tuberculosis [5].

Differential diagnosis

Tuberculosis can mimic virtually any GIT disease and should be considered in the differential diagnosis of neoplasms, inflammatory bowel disease, and other infectious conditions, such as yersiniosis, histoplasmosis, actinomycosis, schistosomiasis, ameboma, syphilis, lymphogranuloma venereum, and periappendiceal abscess [5].

Due to the symptom of dysphagia, esophageal mycobacterial infections may also be confused with gastroesophageal reflux disease and, based on endoscopic appearance, with viral lesions such as those caused by CMV, HSV, and EBV, as well as with neoplastic lesions, reinforcing the importance of immunohistochemical testing for accurate differentiation [6].

Histopathologically, the hallmark of tuberculosis is granuloma formation; however, granulomas may also be present in other conditions, including infectious diseases (histoplasmosis, actinomycosis, schistosomiasis, syphilis, lymphogranuloma venereum), inflammatory bowel disease, vasculitides, and other disorders [5].

Complications

Gastrointestinal involvement by mycobacterial infections may be associated with complications such as aspiration pneumonia, massive gastrointestinal bleeding, fistula formation, traction diverticula, esophageal strictures, perforation, peritonitis, abscesses, and intestinal obstruction [5,6]. In such cases, surgical management may be required as an adjunct to antituberculous therapy [6,28].

In our case series, some of these complications were observed, including the progression of an ulcerated lesion to a hemorrhagic-base lesion covered by fibrinous exudate with irregular margins following local biopsy, as well as the presence of an esophagomediastinal fistula with purulent discharge.

Treatment

The treatment of tuberculous mycobacteriosis may vary according to the reference used for each location, but in general, it consists of combination therapy with rifampicin (R), isoniazid (H), pyrazinamide (Z), and ethambutol (E) [5,6,20]. In Brazil, treatment consists of two phases, intensive and maintenance [25]. The intensive phase for adults consists of four drugs (RHZE) and, for children, three drugs (RHZ), while maintenance consists of two drugs (RH) [25]. The intensive or attack phase lasts two months, and the maintenance phase varies from four to 10 months, depending on the form of presentation, with extrapulmonary meningeal and osteoarticular forms classically treated with 10 months of maintenance [25]. 

In PLWHA, there is still concern about immune reconstitution inflammatory syndrome (IRIS) when starting ART early in the concomitant treatment of tuberculosis [29]. However, such early initiation contributes to a decrease in mortality, except for meningeal tuberculosis [29].

For NTM, the studies cite different treatments, which can be guided by the sensitivity profile and the specific microorganism isolated, generally including drugs such as rifampicin, rifabutin, macrolides (azithromycin or clarithromycin), amikacin, ethambutol, and/or levofloxacin [1,3,16]. 

## Conclusions

Mycobacteriosis of the GIT represents significant morbidity and mortality, especially in HIV-positive individuals and immunosuppressed individuals in general. In this sense, the endoscopic approach provides a valuable diagnostic tool, and it is essential to disseminate knowledge of endoscopic patterns of involvement, especially for professionals working outside reference centers for such patients. Due to the nonspecificity of clinical presentation and endoscopic patterns, it is crucial to maintain a high index of suspicion and pursue microbiological diagnosis. In addition, it is worth emphasizing the importance of increased investment in improving the sensitivity and specificity of diagnostic tests, as well as in more effective and convenient therapies, given the importance of early diagnosis and treatment initiation in preventing morbidity and mortality from mycobacteriosis.
